# Effect of clinical whole exome sequencing in aetiological investigation and reproductive risk prediction for a couple with monogenic inherited diseases

**DOI:** 10.3389/fgene.2024.1364769

**Published:** 2024-05-30

**Authors:** Yanan Wang, Yuqiong Chai, Jieqiong Wang, Mingya Gao, Weiwei Zang, Yujie Chang

**Affiliations:** Department of Genetics and Prenatal Diagnosis, Luoyang Maternal and Child Health Hospital, Luoyang, China

**Keywords:** clinical whole exome sequencing, short stature, SHOX gene, cataracts, CRYBB3 gene

## Abstract

**Objective:**

To determine the genetic causes of monogenic inherited diseases in a couple using clinical whole exome sequencing (WES) and advise on their reproductive choices.

**Methods:**

WES was applied to a couple seeking reproductive advice, the female with short stature and the male with congenital cataracts.

**Results:**

(1) The woman exhibited a 13.8 Kb heterozygous deletion at chrX: 591590–605428 (hg19). This region corresponds to exons 2–6 of the short-stature homeobox-containing (*SHOX*) gene (NM000451). Associated diseases involving the *SHOX* gene range from severe Leri–Weill dyschondrosteosis to mild nonspecific short stature. Meanwhile, further validation using a quantitative reverse transcription polymerase chain reaction assay confirmed the heterozygous deletion of the *SHOX* gene in the proband, as well as other family members with similar clinical characteristics (the proband’s mother, aunt, and cousin). Multiple pathogenic reports of this variant have been included in the HGMD database. Per the American College of Medical Genetics and Genomics (ACMG) classification criteria, this deletion is classified as pathogenic. (2) For the male patient, a heterozygous variant was detected in the *CRYBB3* gene: NM004076: c.226G>A (p.Gly76R). Variants in the *CRYBB3* gene can cause Cataract 22 (OMIM: 609741). At present, this variant locus is not included in databases such as the gnomAD, while both SIFT and PolyPhen2 deem this locus ‘damaging’. Moreover, further validation by Sanger sequencing confirmed that the variant was inherited from the male patient’s mother, who also had cataracts. According to ACMG standards and guidelines, the c.226G>A (p.Gly76Arg) variant in the *CRYBB3* gene is classified as having ‘uncertain significance’.

**Conclusion:**

WES identified pathogenic variants in both individuals, suggesting a 25% chance of a healthy child naturally. Third-generation assisted reproductive techniques are recommended to minimize the risk of affected offspring.

## Patient information

In April 2022, Luoyang Maternal and Child Health Hospital admitted and treated a 33-year-old female with short stature and a 35-year-old male with congenital cataracts for clinical phenotype analysis and the exploration of their genetic aetiology. Following on, guidance and recommendations were provided regarding their reproductive risks.

### Clinical data

**FIGURE 1:**
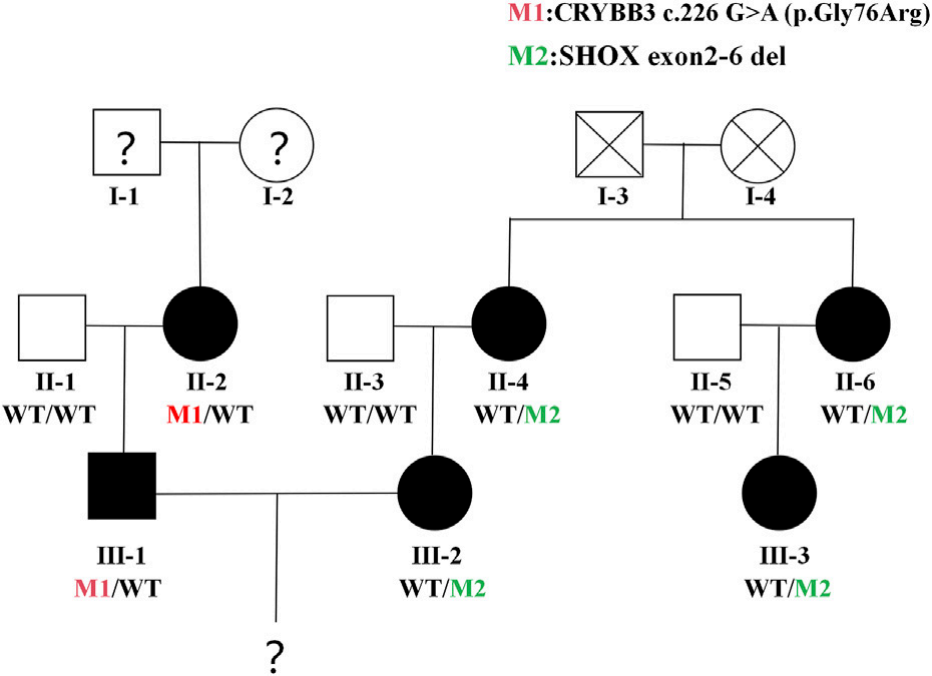
Family Tree. Orange circles or squares = patients in the husband’s family; black circles = patients in the wife’s family. Question marks = data about this family member is described verbally by others, which may not be accurate.

The female patient (wife) was 33 years old and presented with short stature (1.4 m). The patient reported that her mother, aunt, and cousin were also characterised by short stature (1.4–1.5 m); her father was of normal height, leading to an initial suspicion of a bone-dysplasia-related disorder. The male patient (husband) was 35 years old and had congenital cataracts in both eyes. His mother also had cataracts; his father was unaffected, suggesting a maternal inheritance pattern (see Figure 1). This study passed an ethical review and was approved by the ethics committee of our hospital (approval no. LYFY-YCCZ-2023010). Written informed consent was obtained from the patients for the genetic testing of blood samples.

### DNA purification and genetic testing

Genomic DNA was extracted from the peripheral blood samples of the patients and their family members using the QIAamp DNA Blood Mini Kit (QIAGEN, Germany). The genomic DNA was fragmented using an ultrasonicator, with library preparation and capture hybridisation performed using the Agilent Human All 60M Kit (Agilent Technologies, United States of America). Sequencing was performed using the Illumina HiSeq 4000 high-throughput sequencer (Illumina, United States of America). Data processing was conducted to obtain data on variant loci in the targeted regions. An analysis of protein function damage was conducted using software such as SIFT (http://sift.jcvi.org), PolyPhen-2 (http://genetics.bwh.harvard.edu/pph2/index.shtml), and MutationTaster (https://www.mutationtaster.org), based on the raw data. Variant loci requiring further validation were selected based on data, such as the mode of genetic inheritance, clinical symptoms, variant frequency, and sequencing depth. The pathogenicity of the variant loci was analysed and annotated according to the classification criteria and guidelines for genetic variants established by the American College of Medical Genetics and Genomics (ACMG). Moreover, Sanger sequencing was performed to validate the suspected variant loci in the patients and their family members using the ABI3730xl sequencer (Applied Biosystems, United States of America). The sequencing results were compared with DNA sequences published on the Ensemble website using the Snap Gene Viewer software.

## Results

For the female patient, a heterozygous deletion variant of approximately 13.8 Kb was detected at the genomic position chrX:591590–605428 (hg19). This region contains exons 2–6 of the short-stature homeobox-containing (*SHOX*) gene. Quantitative reverse transcription polymerase chain reaction validation was performed using primers designed for exons 2–6 of the *SHOX* gene, and the same heterozygous deletion was detected in the female patient, her mother, aunt, and cousin. Multiple pathogenic reports of this variant have been included in the HGMD database. According to the ACMG guidelines, this deletion is classified as pathogenic.

**FIGURE 2:**
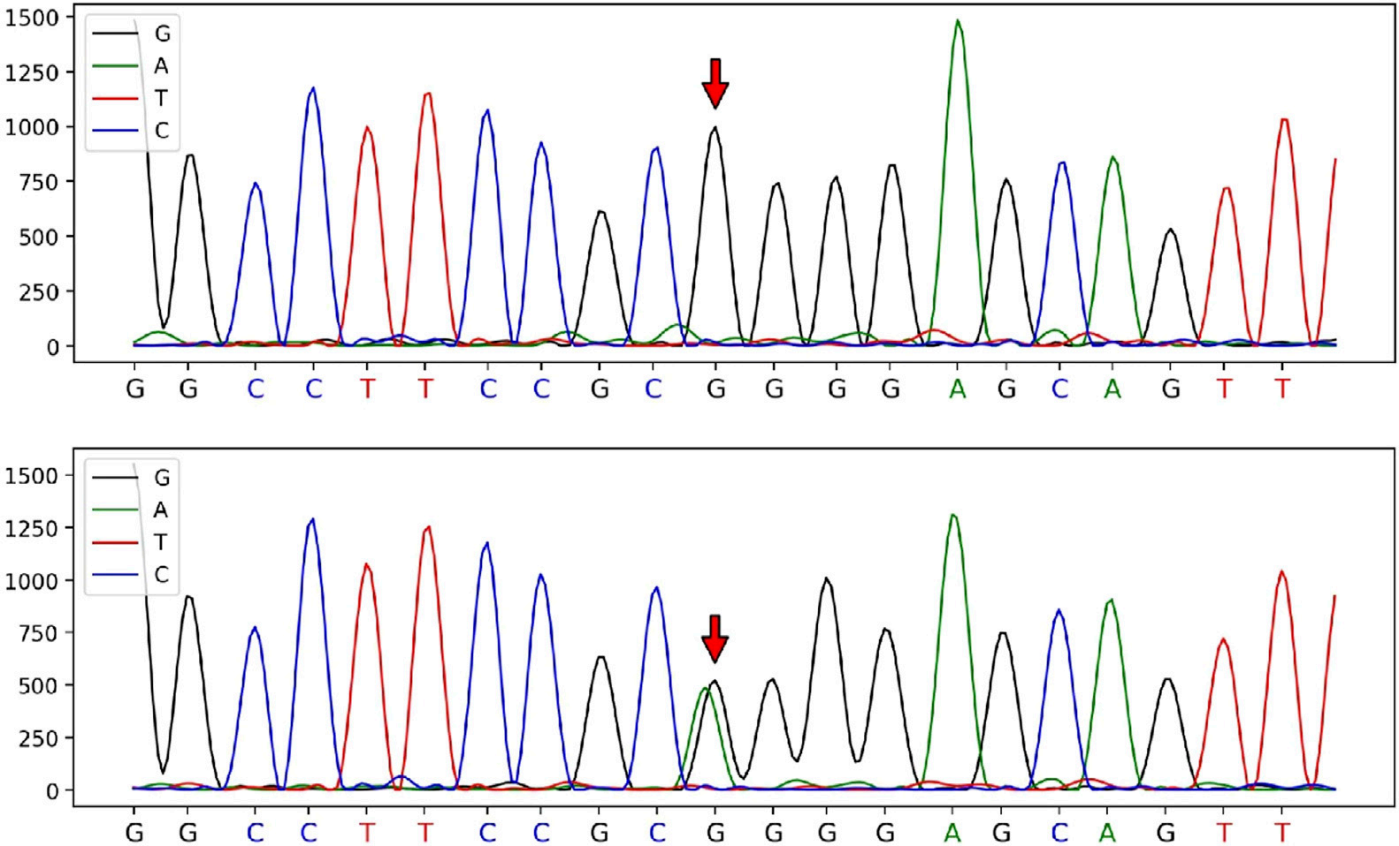
Sanger sequencing peak diagram of the c.226 locus in the *CRYBB3* gene of the husband and his parents.

**FIGURE 3:**
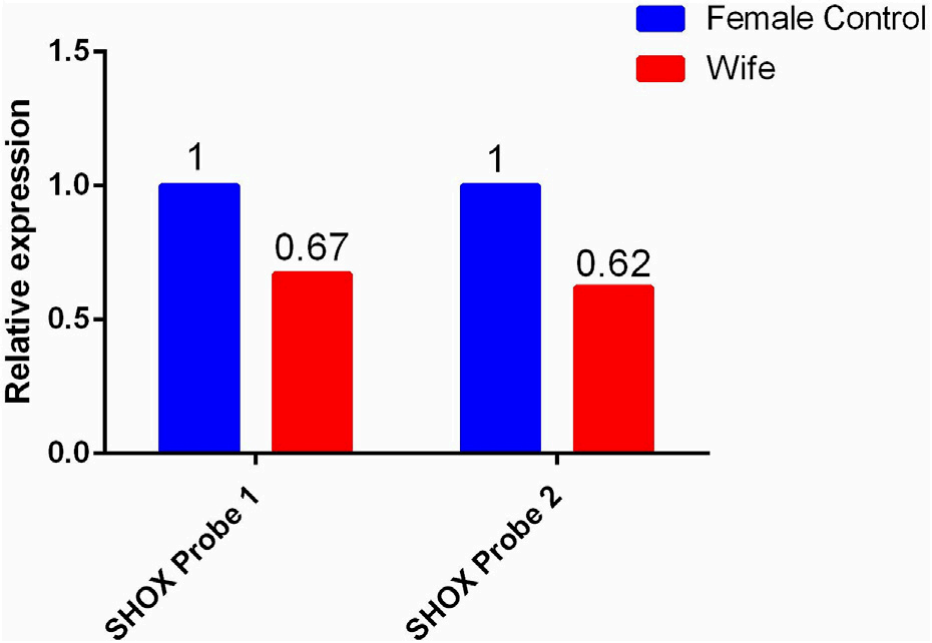
qPCR validation results of the heterozygous deletion in exons 2-6 of the SHOX gene in the wife and her blood relatives.

In contrast, a heterozygous variant in the *CRYBB3* gene: NM_004076:c.226G>A (p.Gly76R) was detected in the male patient. This variant is rare, with a frequency of 0 in the general population based on data from the 1,000 Genomes, ExAC, and gnomAD databases, indicating pathogenic moderate (PM2) evidence according to ACMG guidelines. Sanger sequencing showed that the variant had been inherited from the male patient’s mother and was not detected in other unaffected family members, demonstrating pathogenic supporting (PP1) evidence of familial co-segregation. Additionally, predictions using the SIFT and PolyPhen-2 software indicated a ‘damaging’ effect (PP3). The phenotype of the variant carrier was highly consistent with the monogenic genetic disorder cataract (PP4). Although the male patient’s heterozygous *CRYBB3* variant is currently classified as a variant of uncertain significance, based on the evidence (PM2+PP1+PP3+PP4), the fact that his mother also had cataracts suggests that it was most likely pathogenic, albeit not with certainty. According to ACMG standards and guidelines, the variant c.226G>A (p.Gly76Arg) in the *CRYBB3* gene is classified as having ‘uncertain significance’ (see Figures 2, 3).

## Discussion

### *SHOX* gene-associated diseases

Numerous studies employing whole exome sequencing (WES) have been instrumental in gaining a better understanding of the genetic underpinnings of short stature. Liu et al. made a notable contribution in this area by identifying a novel heterozygous variant in the *SHOX* gene, which was found in a family exhibiting idiopathic short stature, echoing the findings of this study (Liu et al., 2023). Furthermore, research by Hauer et al. underscores the clinical relevance of WES, demonstrating its efficacy in diagnosing conditions like Leri–Weill dyschondrosteosis (LWD) and reinforcing the crucial role of genetic counselling for affected families (Hauer et al., 2017).

Located in the pseudoautosomal region of the X chromosome, the *SHOX* gene [OMIM 312865] is one of the essential genes that regulate cartilage development. It is a dosage-sensitive gene and can lead to a range of height-related disorders, from mild non-specific short stature to severe LWD (Binder et al., 2005). When patients are classified as having idiopathic short stature (ISS) or diagnosed with LWD, Langer mesomelic dysplasia, or Madelung’s deformity, it is typically first considered whether abnormalities are present in the *SHOX* gene (Capkova et al., 2020). There are two main molecular defects in the *SHOX* gene, the most common being large deletions of entire the *SHOX* gene (Capkova et al., 2020), followed by small locus variants, including substitutions, insertions, and deletions of bases (Gürsoy et al., 2020).

In the current case, the female patient was detected as having had a heterozygous deletion of exons 2–6 of the *SHOX* gene, and no other height-related pathogenic variants were found. Combined with the results of the patient’s mother, aunt, and cousin, who also suffered short stature and were found to have the same variant, it is reasonable to accept this heterozygous deletion as having been the pathogenic factor leading to familial idiopathic short stature in the female patient’s family.

### *CRYBB3* gene-associated diseases

Recent advancements in WES have significantly contributed to our understanding of congenital cataracts, particularly through the identification of variants in crystallin genes. A study conducted by Yu et al. was pivotal in this regard, revealing 6 β-crystallin gene variants associated with congenital cataracts in Chinese families, thereby enriching our knowledge of the genetic variability underlying these conditions (Yu et al., 2021). Similarly, Wang et al. discovered novel variants in the *CRYBB3* gene, aligning with our observations of the c.226G>A heterozygous variant in the *CRYBB3* gene and its association with autosomal dominant congenital cataracts (Wang et al., 2021).

Congenital cataracts are defined as lens opacities at birth or in early childhood, with an overall prevalence of 0.32–22.9/10,000 children (Li et al., 2020). It accounts for 21.3% of childhood blindness cases worldwide, with a higher incidence in low- and middle-income countries (Yekta et al., 2022). The factors causing congenital cataracts are complex, including genetics, metabolism, trauma, and infection, with genetic factors accounting for up to 25% (Shiels et al., 2010). Currently, more than 100 pathogenic genes and 200 loci have been identified as being associated with hereditary cataracts, with most variants being autosomal dominant (AD) and a few autosomal recessive (AR). The Cat-Map website (http://cat-map.wustl.edu/) provides a comprehensive summary of the genetic information mentioned above (Shiels et al., 2010).

Studies have shown that approximately 50% of hereditary cataracts are caused by variants in crystallin genes (Bari et al., 2019). Crystallins include two superfamilies, α-crystallins and βγ-crystallins, where β-crystallins consist of similar N-terminal and C-terminal domains separated by a short linker peptide. Different β-crystallins can interact with each other to form oligomers of different sizes or interact with other lens proteins, which are believed to be crucial for keeping the lens of the eye transparent (Bari et al., 2019). Additionally, different variants in β-crystallin genes can cause different types of cataracts, including variants in the β-B3 crystallin gene (*CRYBB3*; OMIM 123630), which cause Cataract 22 (OMIM 609741). As of 3 October 2022, the Cat-Map website has included data on 10 *CRYBB3* gene variant loci, including 7 AD genetic loci: c.75 + 1G>A (Chen and Zhu, 2021), c.224G>A (Rodríguez-Solana et al., 2023), c.466G>A (Taylan Sekeroglu et al., 2020), c.467G>A (Zin et al., 2021), c.531G>T (Fernández-Alcalde et al., 2021), c.581T>A (Xu et al., 2021), and c.634T>C (Fan et al., 2020), as well as 1 AR genetic locus, c.493G>C (Iqbal et al., 2020).

In the current case, the c.226G>A heterozygous variant in the *CRYBB3* gene was detected in both the husband and his mother, which, despite being classified by the ACMG as ‘clinically unknown’, we believe is highly likely to be a pathogenic locus. The reasons for this are as follows: ① This locus is located in the ‘Greek key’ domain, which is extremely conserved in the βγ-crystallin superfamily, and most of the known pathogenic loci found to date are concentrated in this domain (Li et al., 2020). ② The variant is classified as ‘damaging’ by multiple variant prediction software based on different principles. ③ Most known pathogenic loci in *CRYBB3* are known to be inherited in an AD pattern. However, the fact that the female patient (II-2) reported her parents (I-1 and I-2) as not having had cataracts does not necessarily rule out an AD inheritance in this case. The *CRYBB3* variant could have arisen *de novo* in the wife or could have been transmitted from an unaffected parent carrying the variant in their germ cells. Additionally, an AR or X-linked recessive inheritance pattern cannot be excluded based on the available information. However, further population evidence and functional experimental verification are still needed in the future.

The findings of this study provide valuable assistance for the couple in terms of having children, indicating only a 25% probability of naturally conceiving a healthy child without *SHOX* gene defects or Cataract 22. The husband carries a heterozygous variant in the *CRYBB3* gene (*CRYBBM/CRYBBN*), while the wife has a heterozygous deletion in the *SHOX* gene (*SHOXM/SHOXN*). There is a 25% chance of having a child with neither variant (*SHOXN/CRYBBN*), a 25% chance of having a child with both variants (*SHOXM/CRYBBM*), and a 50% chance of having a child heterozygous for either the *SHOX* or *CRYBB3* variant. Therefore, the couple has a 1 in 4 chance of conceiving a healthy child without any of the parental variants through a natural pregnancy. Therefore, it is recommended that the couple seek help from third-generation assisted reproductive techniques to screen healthy embryos for pregnancy and avoid the birth of affected children. Moreover, since WES narrows down the range of detected genes (i.e., focusing only on clinically established pathogenic genes) compared with whole-genome sequencing (WGS), its test results are more targeted, easier to interpret, and relatively affordable, making it a better choice for non-wealthy families.

## Data Availability

The data presented in the study are deposited in the Genome Sequence Archive for Human repository, accession number HRA006933.
